# Effects of Gold Nanoparticles Functionalized with *Cornus mas* L. Fruit Extract on the Aorta Wall in Rats with a High-Fat Diet and Experimental-Induced Diabetes Mellitus—An Imaging Study

**DOI:** 10.3390/nano13061101

**Published:** 2023-03-19

**Authors:** Remus Moldovan, Daniela-Rodica Mitrea, Adrian Florea, Luminiţa David, Laura Elena Mureşan, Irina Camelia Chiş, Şoimița Mihaela Suciu, Bianca Elena Moldovan, Manuela Lenghel, Liviu Bogdan Chiriac, Irina Ielciu, Daniela Hanganu, Timea Bab, Simona Clichici

**Affiliations:** 1 Department of Physiology, Iuliu Hatieganu University of Medicine and Pharmacy, 1–3 Clinicilor Street, 400006 Cluj-Napoca, Romania; 2 Department of Cell and Molecular Biology, Iuliu Hatieganu University of Medicine and Pharmacy, 6 Pasteur Street, 400349 Cluj-Napoca, Romania; 3 Research Center for Advanced Chemical Analysis, Instrumentation and Chemometrics, Faculty of Chemistry and Chemical Engineering, Babes-Bolyai University, 11 Arany Janos Street, 400028 Cluj-Napoca, Romania; 4 Raluca Ripan Institute of Research in Chemistry, Babes-Bolyai University, 30 Fantanele Street, 400294 Cluj-Napoca, Romania; 5 Radiology Department, Iuliu Hatieganu University of Medicine and Pharmacy, 1–3 Clinicilor Street, 400006 Cluj-Napoca, Romania; 6 Medical Biophysics, Iuliu Hatieganu University of Medicine and Pharmacy, 6 Pasteur Street, 400394 Cluj-Napoca, Romania; 7 Faculty of Physics, Babeş-Bolyai University, 1 Mihail Kogalniceanu Street, 400084 Cluj-Napoca, Romania; 8 Department of Pharmaceutical Botany, Faculty of Pharmacy, Iuliu Hatieganu University of Medicine and Pharmacy, 23 Gheorghe Marinescu Street, 400010 Cluj-Napoca, Romania; 9 Department of Pharmacognosy, Faculty of Pharmacy, University of Medicine and Pharmacy Iuliu Hatieganu, 400000 Cluj-Napoca, Romania; 10 SC PlantExtrakt SRL, Radaia, 407059 Cluj, Romania

**Keywords:** aorta, gold nanoparticles, *Cornus mas*, antioxidants, endothelium

## Abstract

Diabetes mellitus and high-fat diets trigger the mechanisms that alter the walls of blood vessels. Gold nanoparticles, as new pharmaceutical drug delivery systems, may be used in the treatment of different diseases. In our study, the aorta was investigated via imaging after the oral administration of gold nanoparticles functionalized with bioactive compounds derived from *Cornus mas* fruit extract (AuNPsCM) in rats with a high-fat diet and diabetes mellitus. Sprague Dawley female rats that received a high-fat diet (HFD) for 8 months were injected with streptozotocin to develop diabetes mellitus (DM). The rats were randomly allocated into five groups and were treated, for one additional month with HFD, with carboxymethylcellulose (CMC), insulin, pioglitazone, AuNPsCM solution or with *Cornus mas* L. extract solution. The aorta imaging investigation consisted of echography, magnetic resonance imaging and transmission electron microscopy (TEM). Compared to the rats that received only CMC, the oral administration of AuNPsCM produced significant increases in aorta volume and significant decreases in blood flow velocity, with ultrastructural disorganization of the aorta wall. The oral administration of AuNPsCM altered the aorta wall with effects on the blood flow.

## 1. Introduction

The obesity–diabetes mellitus–hypertension triad represents a well-known pathological interconnection that still requires studies of the involved factors, intricate mechanisms and possible treatments.

Data in the literature present a high-fat diet (HFD) as the promoter of heart, kidney and liver impairments, diabetes mellitus (DM) or atherosclerosis development, among other tissue alterations [1].

Diabetes mellitus type 2 evolves through different mechanisms (oxidative stress produced by chronic hyperglycemia, insulin resistance, polygenic defects, environmental trigger factors, etc.) [2] and requires specific treatment approaches. Numerous researchers studied the effects of different natural extracts (*Aloe vera*, *Zingiber officinale*, *Tabernaemontana divaricata*, etc.) on the pathophysiological mechanisms of DM, showing beneficial effects on hyperglycemia [3,4,5] and on diabetes-related cardiovascular impairments [6].

New pharmaceutical drug delivery systems, including nanoparticles of different structures, dimensions, chemical and electrical properties, were developed to improve the treatment of diseases [7]. Gold nanoparticles (AuNPs) are considered biocompatible and stable delivery systems [8], but several studies have presented the noxious potential of AuNPs functionalized with different natural extracts on the liver [9], DNA [10] or with conflicted results in the cardiovascular system [11].

Our study evaluated rats with chronic HFDs and DM, treated with *Cornus mas* L. extract (CM) or gold nanoparticles functionalized with *Cornus mas* (AuNPsCM) to evaluate modifications in the aorta wall using imaging techniques.

The present study is part of a larger project that started with the idea that gold nanoparticles could improve the delivery of the *Cornus mas* L. extract in the vessel wall, to prevent or even to solubilize the atherosclerotic plaques that occur with a prolonged, high-fat diet with or without experimental-induced diabetes mellitus.

## 2. Materials and Methods

### 2.1. Fruit Extract Preparation and Characterization

All chemicals used to obtain fruit extract and synthesize the gold nanoparticles were purchased from Merck (Darmstadt, Germany).

The Cornelian cherries (bought from the Central Market of Cluj-Napoca in August 2021 and kept frozen until use) were crushed and mixed with food-grade acetone (in a 1:5 ratio). After stirring for 1 h at ambient temperature, the mixture was vacuum-filtrated, and the acetone was totally removed using low-pressure distillation. The biological activity of the resulting concentrated fruit extract was determined and used to synthesize the gold nanoparticles. The Cornelian cherry extract was characterized in terms of total phenolic content, determined using the Folin–Ciocalteu assay [12] with minor modifications [13]. Thus, to a mixture of Folin-Ciocalteu reagent (100 µL) and Cornelian cherry fruit extract (10 µL), Na_2_CO_3_ (80 µL) were added. After 2 h of storage in a dark at room temperature, the absorbance of the solution was recorded at 765 nm. Using a calibration curve (solutions in the range of 0.025–0.15 mg/mL, R^2^ = 0.9986), the total phenolic content of the fruit extract was expressed as milligrams of gallic acid equivalents (GAE)/mL extract [12,14,15].

### 2.2. High-Performance Liquid Chromatography (HPLC)

The LC/MS analysis was performed on a Shimadzu Nexera I LC/MS—8045 (Kyoto, Japan) HPLC system equipped with a quaternary pump and autosampler, respectively, an ESI probe and quadrupole rod mass spectrometer. The separation was carried out on a Luna C18 reversed-phase column (150 mm × 4.6 mm × 3 mm, 100 Å) from Phenomenex (Torrance, CA, USA). The column was maintained at 40 °C during the analysis. The mobile phase was represented using a gradient made from methanol (Merck, Darmstadt, Germany) and ultra purified water prepared with a Simplicity Ultra Pure Water Purification System (Merck Millipore, Billerica, MA, USA). Formic acid (Merck, Darmstadt, Germany) was used as an organic modifier. The methanol and the formic acid were of LC/MS grade. A flow rate of 0.5 mL/minute was used. The total time of an analysis was 35 min. The detection was performed on a quadrupole rod mass spectrometer operated with electrospray ionization (ESI), both in negative and positive multiple reaction monitoring (MRM) ion mode. The interface temperature was set at 300 ºC. Nitrogen was used at 35 psi for vaporization and as drying gas, respectively, at 10 L/min. The capillary potential was set at +3000 V.

The identification was performed using comparison of retention times, MS spectra and its transitions between the separated compounds and standards. The identification and quantification were conducted based on the main transition from the MS spectra of each compound. For quantification purposes, the calibration curves were determined. The injected volume for each standard at each concentration was 1 µL [14,15]. All compounds used were purchased from Phytolab, Vestenbergsgreuth, Germany.

### 2.3. Gold Nanoparticles Synthesis, Characterization and Tissue Determinations

Gold nanoparticles were obtained using tetrachloroauric acid as the source of gold ions and the Cornelian cherry fruit extract as the source of reducing and capping bioactive compounds. Thus, the alkalinized fruit extract (brought at pH = 7.5 using a 0.1 M solution of NaOH) was slowly added to a boiling 1 mM solution of HAuCl_4_ (in a 1:4 ratio) and the resulted mixture was stirred at room temperature. After 30 min, the change of the color from faint pink to red–purple confirmed the formation of colloidal gold. The colloidal solution was subjected to centrifugation at 14,000 rpm for 30 min and the resulting AuNPs were washed twice with bidistilled water and air-dried. UV-Vis spectroscopy (using a Perkin-Lambda 25 double beam spectrometer, band width 1 nm, minimum spectral resolution 0.5 nm, wavelength accuracy ±0.1 nm) and transmission electron microscopy (using a Hitachi Automatic H-7650 microscope) was used to characterize the obtained gold nanoparticles. ImageJ software was used for automatic particle counting and size determination, 100 nanoparticles were considered.

The zeta potential of the gold nanoparticles was determined through microelectrophoresis using a DLS instrument with a He–Ne laser (633 nm) and an avalanche photodiode detector.

The level of the gold nanoparticles in the aorta wall was determined with ICP–OES (inductively coupled plasma–optical emission spectrometry) using a Perkin Elmer OPTIMA 2100 DV spectrometer, following the method that was described in our previous article [16].

### 2.4. Animals

Sprague Dawley adult female rats were used to investigate the effects of gold nanoparticles functionalized with *Cornus mas* L. extract (AuNPsCM) as treatment after prolonged high-fat diet and experimental-induced diabetes mellitus. The animals (35 rats), 3 months old with body weight of 300 ± 10 g, were brought from Cantacuzino National Medico-Military Institute for Research and Development, Bucharest, Romania. The rats were hosted in cages in standard environmental conditions (temperature 21 ± 2 °C, relative humidity 55% ± 5) and were nourished exclusively with standardized rich lipid food. After 9 months of high-fat diet (HFD), the rats increased their body weight at 600 ± 10 g. The access to filtered tap water was ad libitum, like the access to the same type of feed that was administered by gavage every day. The study had the approval of the Ethics Committee of the Iuliu Hatieganu University of Medicine and Pharmacy (no. 158/11.03.2019) according to the Directive 86/609/EEC.

### 2.5. High-Fat Diet (HFD)

The lipid-rich diet used for animal feeding was purchased from the Cantacuzino National Medico-Military Institute for Research and Development, Bucharest, Romania. The high-fat food was administered by gavage, bringing an additional 45% level of energy. The composition of the diet was described in our preliminary study [16].

### 2.6. Diabetes Mellitus Induced by Streptozotocin Administration

During the last 3 days of the 8th month of the experiment, diabetes mellitus was induced in all rats in the following manner: streptozotocin was injected intraperitoneally, 30 mg/kg, 2 times, 72 h apart.

### 2.7. Experimental Design

In the study, 35 rats were randomly allocated into 5 groups (n = 7) with high-fat diet (HFD) for the entire duration of the experiment. At the beginning and at the end of the experiment, the rats were weighed. After 33 weeks of HFD, diabetes mellitus was induced in all rats and the treatment was introduced at 3 days after the streptozotocin administration when all rats had glycemia above 250 mg/dL. The diabetic rats were treated daily, between 8 a.m. and 9 a.m., for one month (the 9th month of the experiment with HFD) as follows: *CMC group*: 0.6 mL/day of 1% carboxymethylcellulose solution, through gavage; *Insulin group*: 0.1 mg/kg of insulin, subcutaneous injection; *Pioglitazone group*: 0.6 mL/day of pioglitazone solution, 10 mg/kg, through gavage; *AuNPsCM group*: 0.6 mL/day of gold nanoparticles functionalized with *Cornus mas* L. extract (260 μg AuNPs/kg/day), through gavage; *CM group*: 0.6 mL/day of *Cornus mas* L. extract solution (30 mg/kg/day of polyphenols), through gavage.

During the last day of the experiment, ultrasound and MRI scans were performed. Ketamine 10% (5 mg/100 gbw) and xylazine hydroxychloride 2% (100 mg/100 gbw) were used to induce deep anaesthesia in rats and descending thoracic aortas were collected for transmission electron microscopy investigation.

### 2.8. Ultrasound (US) Evaluation

A 2D Doppler transthoracic echocardiogram was performed to determine the blood flow speed and the aorta caliber, using a Sonotouch Tablet System (Ultrasonix Medical Corporation, Richmond, BC, Canada). The animals were sedated throughout the whole procedure. The system included a MS250 transducer that used a frequency of 24 MHz with harmonics and 16 MHz with Doppler. The transducer was placed on a system with linear positioning, which allowed it to pan across the scanning area. The Doppler incidence angle was 51° with a maximal individual correction of 60°, conducted for each rat.

### 2.9. IntraGate Flash CINE

The aortas of all rats were scanned. Before scanning, the animals were anesthetized through intramuscular administration of 100 mg/kg ketamine and 50 mg/kg xylazine at a 2:1 ratio. After complete anaesthesia was confirmed, the rats were placed in a ventral decubitus position on the MRI bed and connected to an external ECG device to monitor and synchronize the IntraGate FLASH.

This method provided two core advantages: both a short preparation and examination time. Moreover, it enabled the acquirement of high-resolution images, which in turn, allowed for accurate volumetric measurements and a low error rate in determining aortic functionality.

The MRI model utilized for scanning was BrukerBioSpec 70/16 USR, operated at 7 Tesla, with a dedicated IntraGate FLASH protocol, used to acquire structural images at the T_2_ relaxation time. It was equipped with a superconductive magnet, which functioned at a temperature of 4.2 Kelvin, with an active diameter of 160 mm, while the gradient unit (BGS 9 HP) offered 90 mm for the radiofrequency (RF) coils used to investigate the experimental animals. The dual resonance frequency was configured for investigations conducted at the 300 MHz mark for hydrogen protons, and, respectively, a varying frequency for the X channel.

**Figure 1:**
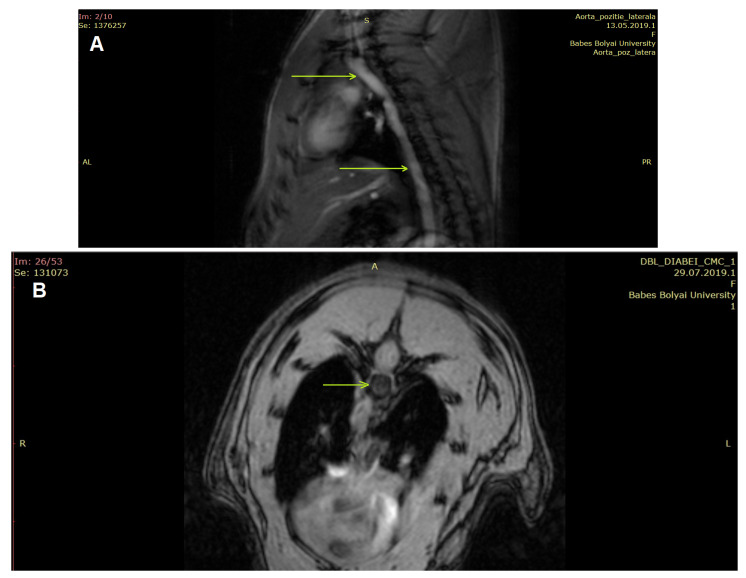
The investigated aorta segment (sagittal section) (**A**) and an example of the sections used for 3D reconstruction (**B**), using the IntraGateFlash CINE scanning technique.

The protocol for the rat aorta geometry study followed a 2D IntraGateTripilot design with a visual field of 6 cm, a section width of 1 mm and an inter-section distance of 2 mm, obtained through a repeating time interval of 200 ms and an ECO time of 3 ms. The tripilot images with a sagittal section were investigated to confirm that the acquired sections included the area of interest, as depicted in Figure 1.

For aorta reconstruction, an IntraGateFlash CINE scanning technique was used—a fast acquiring protocol, with a field of view of 4.20–5.60 cm, axially oriented in a specific way that eased reconstruction, with a section width of 0.8–1 mm and an inter-slice length of 0.4–1.5 mm and a matrix of 256 × 256, which assured a resolution power between 0.0128 and 0.0129 cm/pixel. These adjustments, coupled with a repetition time of 453–511 ms and 45–69 slices, led to a maximal acquirement time of 5 min and 36 s.

The measurements were performed on the descending aorta, 10 mm of its superior part, after the aortic arch.

**Figure 2:**
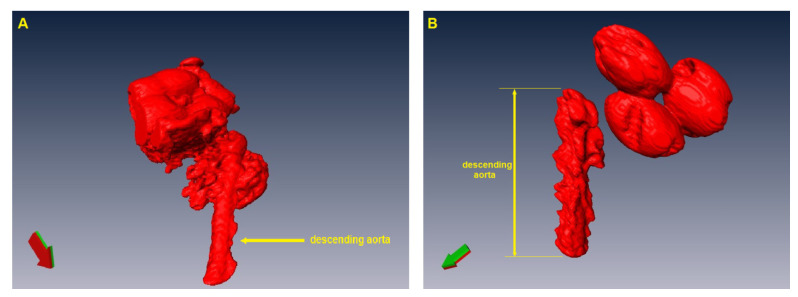
3D reconstruction of rat aorta and heart: (**A**) aorta and heart before the removal of ascending aorta and aortic arch; (**B**) selected descending aorta used for volume evaluation, and the isolated heart.

The images obtained for 3D reconstruction were run through the specialized software AMIRA. After reconstruction, the descending aorta was selected, and the surrounding areas were removed (Figure 2). Following reconstruction, the software automatically calculated the volume of interest according to a table.

### 2.10. Transmission Electron Microscopy (TEM)

For the TEM investigation, the aorta samples were prepared using the method described in our previous article [16]. The aorta sections of 60–80 nm were examined with a JEOL JEM 100CX II transmission electron microscope (JEOL, Tokyo, Japan) and the images were taken with a MegaView G3 camera (EMSIS, Münster, Germany).

### 2.11. Statistical Processing

GraphPad Prism version 5.03 for Windows, GraphPad Software (San Diego, CA, USA) was used to evaluate the modification significance of the measured parameters with a one-way ANOVA followed with the Tukey post-test. The threshold significance level was considered at *p* < 0.05.

## 3. Results

### 3.1. Characterization of Cornus mas L. xtract

The results obtained for the identification and quantification of the polyphenolic compounds from the tested extract are presented in Table 1, together with their retention times and main MS transitions.

### 3.2. Characterization of Gold Nanoparticles Functionalized with Cornus mas L. Phytocompounds

The distribution of gold nanoparticles on the aorta wall was investigated with a finding of 0.038 ± 0.003 mg/g. The synthesized nanoparticles were stable. After 13 zeta runs, the synthesized nanoparticles presented a zeta potential of −33.7 ± 3.03 mV.

The Folin–Ciocalteu method, applied to determine the total phenolic content of the fruit extract, resulted in a value of 0.7437 ± 0.071 mg GAE/mL of fruit extract.

**Figure 3:**
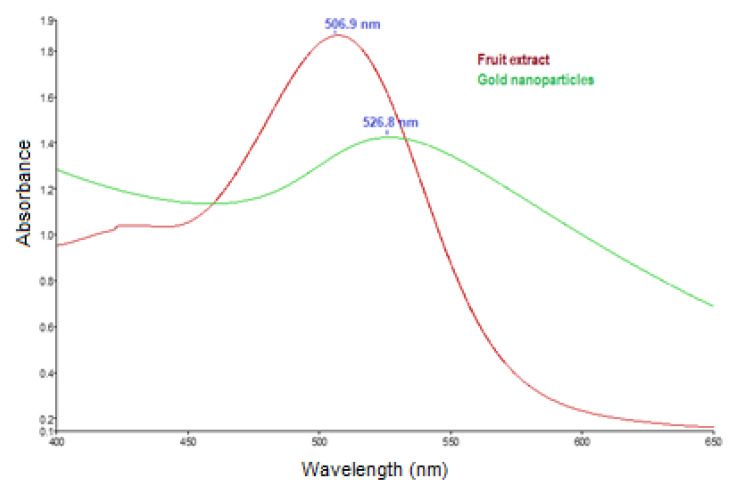
UV-Vis spectra of *Cornus mas* fruit extract and gold nanoparticles.

UV-Vis spectroscopy was used to confirm the formation of the gold nanoparticles through the reduction in gold ions by the bioactive compounds from the Cornelian cherry extract. The UV-Vis spectrum of the Cornelian cherry fruit extract (Figure 3) exhibited a maximum at 507 nm, which is the specific λ_max_ for anthocyanin compounds. In the spectrum of the colloidal gold solution, a maximum at 527 nm could be observed, which is the characteristic value for the surface plasmon resonance of metallic gold [17,18].

**Figure 4:**
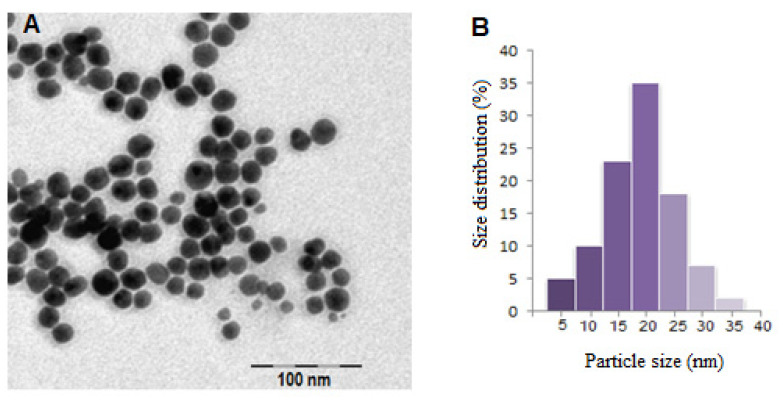
TEM image of gold nanoparticles (**A**) and corresponding particle size distribution (**B**).

The shape and size of the obtained gold nanoparticles were investigated using transmission electron microscopy. Figure 4 shows a TEM image of the investigated AuNPs, which proved that they were spherical and had a mean diameter of 19 nm ± 1.5 nm.

### 3.3. Aorta Investigation

#### 3.3.1. Ultrasound Aorta Examination

**Figure 5:**
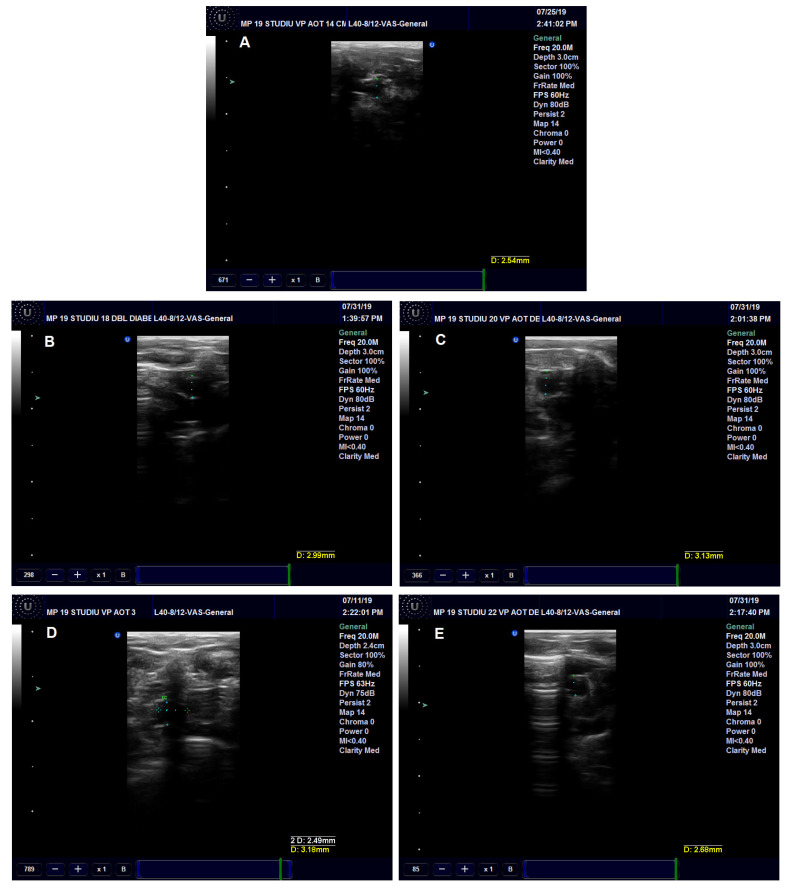
Ultrasound examination of the descending aorta diameter in rats with 9 months of HFD and the last month with experimental-induced diabetes mellitus and treatment: CMC (carboxymethylcellulose) group (**A**), Insulin group (**B**), Pioglitazone group (**C**), AuNPsCM (gold nanoparticles functionalized with *Cornus mas* L. extract) group (**D**) and CM (*Cornus mas*) group (**E**).

**Figure 6:**
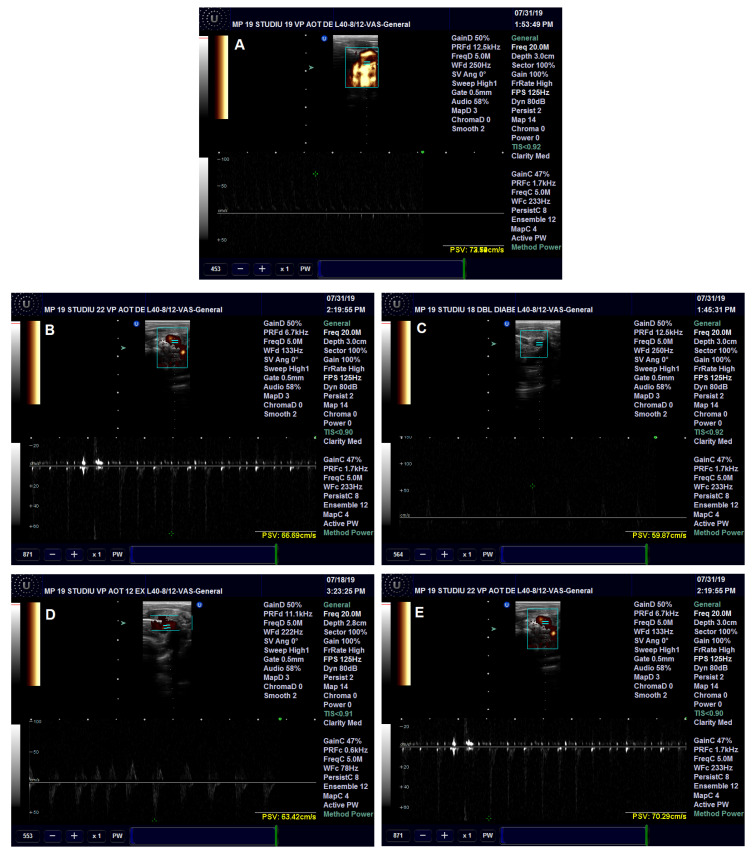
Blood flow in the descending aorta in rats with 9 months of HFD and the last month with experimental-induced diabetes mellitus and treatment: CMC (carboxymethylcellulose) group (**A**), Insulin group (**B**), Pioglitazone group (**C**), AuNPsCM (gold nanoparticles functionalized with *Cornus mas* L. extract) group (**D**) and CM (*Cornus mas*) group (**E**).

**Figure 7:**
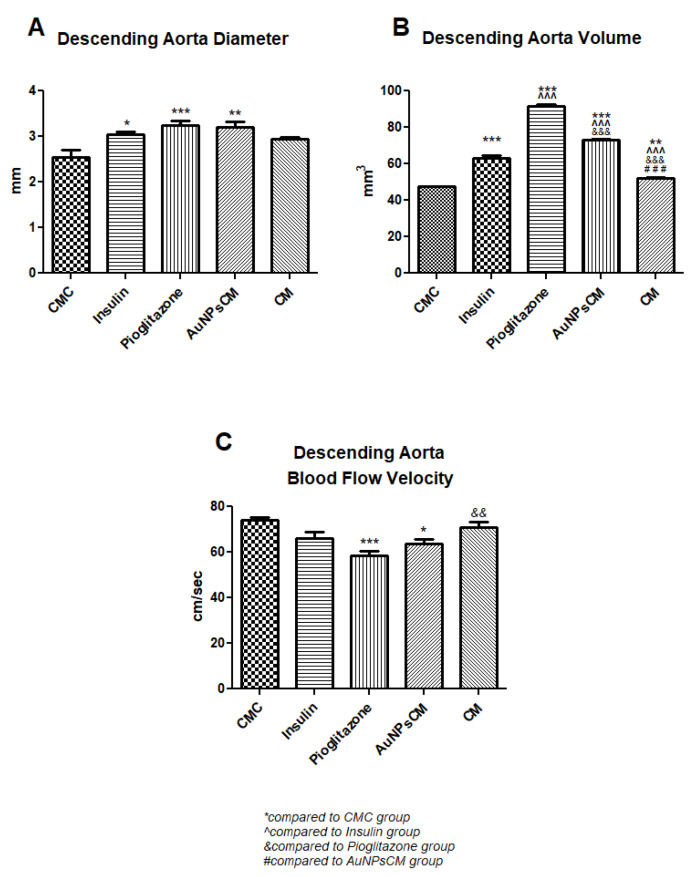
Descending aorta variations of diameter (**A**), volume (**B**) and blood flow velocity (**C**) in rats with 9 months of HFD and the last month with experimental-induced diabetes mellitus and treatment: CMC (carboxymethylcellulose), insulin, pioglitazone, AuNPsCM (gold nanoparticles functionalized with *Cornus mas* L. extract) or CM (*Cornus mas*). The parameters were expressed as means ± SD (* *p* < 0.05, ** *p* < 0.01, *** *p* < 0.001 compared to CMC group; ˆˆˆ *p* < 0.001 compared to Insulin group; && *p* < 0.01, &&& *p* < 0.001 compared to Pioglitazone group; ### *p* < 0.001 compared to AuNPsCM group).

The effects of the administered treatment on the aorta were investigated in the rats with prolonged HFD and experimental-induced DM. The diameter of the rats’ descending aorta was significantly increased in the Insulin (*p* < 0.05), Pioglitazone (*p* < 0.001) and AuNPsCM (*p* < 0.01) groups, compared to the CMC group (Figure 5 and Figure 7A). The blood flow velocity in the descending aorta was correlated with the aorta diameter modifications; significant decreases were recorded in the Pioglitazone (*p* < 0.001) and AuNPsCM (*p* < 0.05) groups, compared to the negative control group. When compared to the positive control Pioglitazone group, the CM group showed significant increases (*p* < 0.01) in the blood flow velocity (Figure 6 and Figure 7C).

#### 3.3.2. IntraGateFlash CINE Scanning Investigation

MRI was used to determine aorta volume. Compared to the CMC group, all of the treated groups presented significant increases in the volume of the aorta: the Insulin, Pioglitazone and AuNPsCM groups (*p* < 0.001); the CM group (*p* < 0.01). In comparison with insulin administration, significant increases (*p* < 0.001) in the volume of the descending aorta was recorded in the groups that received pioglitazone or gold nanoparticles functionalized with *Cornus mas* L. extract, while the treatment with the simple solution of the natural extract significantly (*p* < 0.001) decreased the volume in the descending aorta. The CM group had the smallest increase in descending aorta volume, and, in comparison with the AuNP_S_CM group, the MRI investigation recorded significant decreases (*p* < 0.001) (Figure 7B).

#### 3.3.3. TEM Investigation

**Figure 8:**
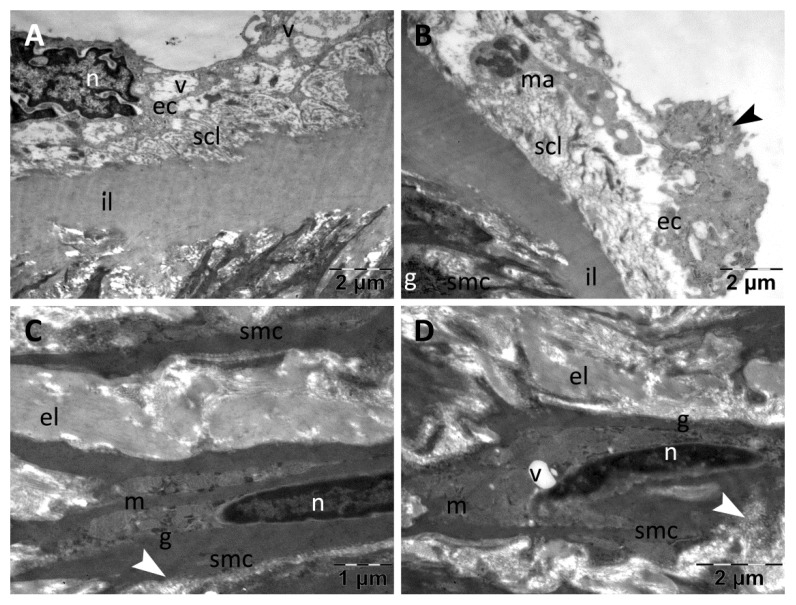
TEM investigation of the descending aorta in rats with HFD for 9 months; the last month with experimental-induced diabetes mellitus and with oral gavage CMC administration showed intima with vacuolated endothelial cells (**A**,**B**), some detached from the subendothelial connective layer into which macrophages were still present (**B**); media showed normal ultrastructural aspect (**C**,**D**), but some vacuoles were noted in the muscle cells (**D**) (arrowhead, vesicles of endocytosis in muscle cells and transcytosis in endothelial cells; ec, endothelial cell; el, elastic lamina; il, inner elastic lamina; g, glycogen; m, mitochondria; ma, macrophage; n, nucleus; scl, subendothelial connective layer; smc, smooth muscle cell; v, vacuole).

In the CMC group, the TEM examination of the aorta samples revealed a normal thickness of intima that was heterogeneous and with fine ultrastructural alterations (Figure 8A,B). The endothelial cells contained numerous and large vacuoles, both around the nucleus and in their extensions (Figure 8A), some of them prominent into the lumen (Figure 8A,B). Rare endothelial cells were partially detached from the subendothelial connective layer, and their numerous transcytosis vesicles indicated intense metabolic activity (Figure 8B). The subendothelial connective layer also had normal thickness but was heterogeneous (Figure 8A) or rarefied (Figure 8B), in many places being visible macrophages that participated int the formation of atherosclerotic plaques. In media, the smooth muscle cells displayed characteristic ultrastructure with many vesicles of endocytosis (Figure 8C,D) and some large cytoplasmic vacuoles (Figure 8D); the extracellular matrix was homogeneous (Figure 8C,D). All elastic laminae were homogeneous, with normal aspect (Figure 8A–D).

**Figure 9:**
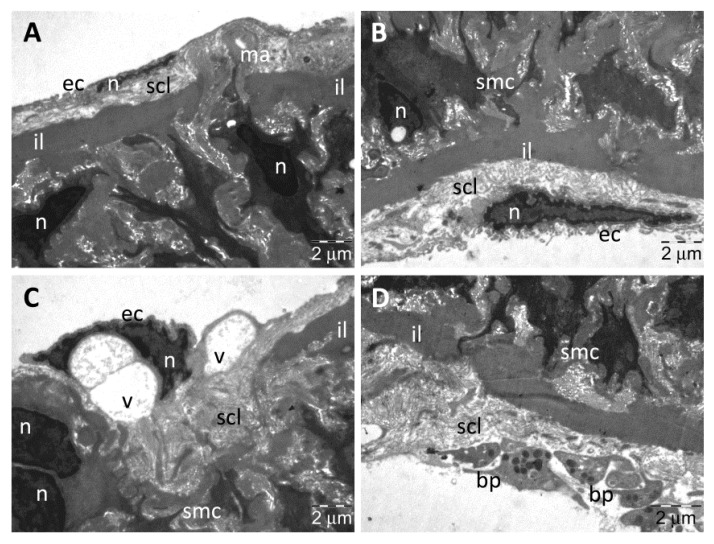
TEM investigation of the descending aorta in rats with HFD for 9 months; the last month with experimental-induced diabetes mellitus and subcutaneous administration of insulin showed altered ultrastructure: thinned intima (**A**,**C**), with large vacuoles (**C**) and endothelial cells removed from the subendothelial layer (**D**), and a subendothelial connective layer with a heterogeneous structure (**A**–**D**); smooth muscle cells with cytoplasmic vacuolations (**A**,**B**), and heterogeneous extracellular matrix with rarefied areas in media (**A**–**D**) (bp, blood platelets; ec, endothelial cell; il, inner elastic lamina; g, glycogen; m, mitochondria; ma, macrophage; n, nucleus; scl, subendothelial connective layer; smc, smooth muscle cell; v, vacuole).

In the aorta of the rats in the Insulin group, the intima showed a thinned and mostly continuous endothelium (Figure 9A–D). The endothelial cells contained many transcytosis vesicles and rare large vacuoles (Figure 9C). In some regions, the endothelial cells were removed from the subendothelial connective layer, and the remaining gaps were filled with aggregated blood platelets forming thin clots (Figure 9D). The subendothelial connective layer had variable thicknesses and heterogeneous structure (Figure 9A–D), with infiltrated macrophages (Figure 9A) and vacuoles (Figure 9C). In media, the smooth muscle cells displayed normal ultrastructure, with the exception of some cytoplasmic vacuolations, most likely enlarged mitochondria (Figure 9A,B), and the extracellular matrix was heterogeneous with rarefied areas. The elastic laminae had a normal, homogeneous aspect (Figure 9A–D).

**Figure 10:**
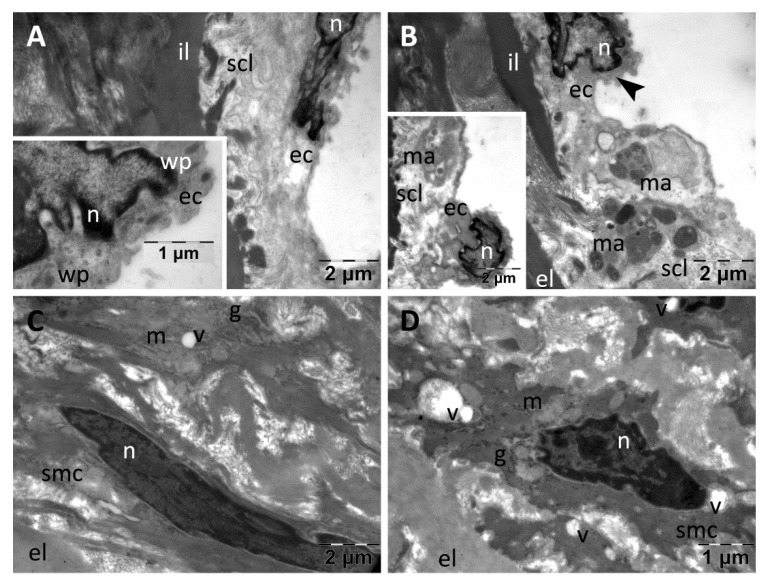
TEM investigation of the descending aorta in rats with HFD for 9 months; the last month with experimental-induced diabetes mellitus and oral gavage pioglitazone administration showed modified ultrastructure: intima of different thickness, with infiltrated macrophages and prominent endothelial cells containing vesicles of transcytosis, large vacuoles and many Weibel–Palade bodies (**A**,**B**); media with vacuolated smooth muscle cells, and heterogeneous extracellular matrix (**C**,**D**) (arrowhead, vesicles of transcytosis; ec, endothelial cell; el, elastic lamina; il, inner elastic lamina; g, glycogen; m, mitochondria; ma, macrophage; n, nucleus; scl, subendothelial connective layer; smc, smooth muscle cell; v, vacuole, wp, Weibel–Palade bodies).

In the Pioglitazone group, the intima was of a different thickness in the different studied regions, in some cases due to the different sizes of the subendothelial connective layer (Figure 10A) or due to the endothelial cells prominent into the lumen, with numerous macrophages (of atherosclerotic plaques) in the subendothelial connective layer, respectively (Figure 10B). In the cytoplasm of the endothelial cells, many vesicles of transcytosis were noted, as well as rare large vacuoles (Figure 10A,B). The endothelial cells also contained many Weibel–Palade bodies (Figure 10A,B). The media presented smooth muscle cells with characteristic aspect, but with extensive cytoplasmic vacuolation, and the matrix among the cells was heterogeneous with rarefied areas (Figure 10C,D). All elastic laminae were homogeneous (Figure 10A–D).

**Figure 11:**
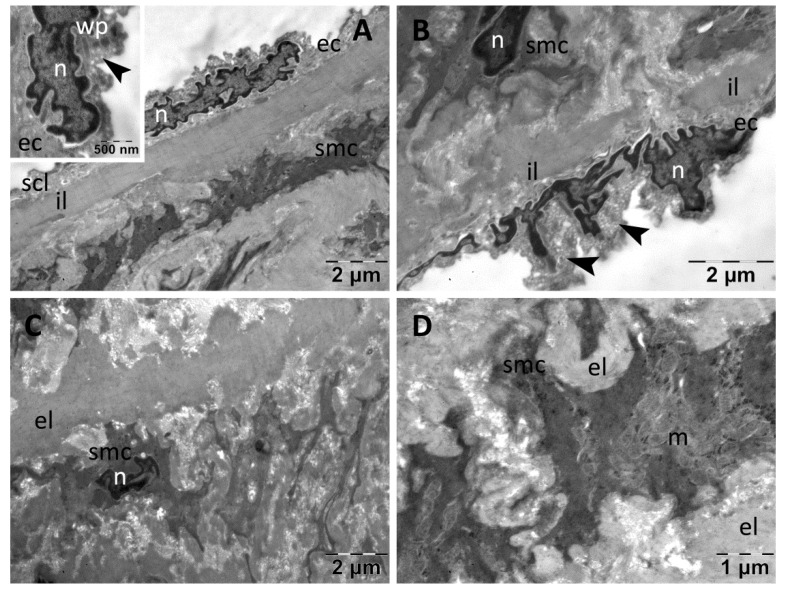
TEM investigation of the descending aorta in rats with HFD for 9 months; the last month with experimental-induced diabetes mellitus and with oral gavage AuNPsCM administration showed impacted ultrastructure: extremely thin intima with thin subendothelial connective layer and prominent endothelial cells containing vesicles of transcytosis and Weibel–Palade bodies (**A**,**B**); media showed normal ultrastructure (**C**,**D**) (arrowhead, vesicles of transcytosis; ec, endothelial cell; el, elastic lamina; il, inner elastic lamina; m, mitochondria; n, nucleus; scl, subendothelial connective layer; smc, smooth muscle cell; wp, Weibel–Palade bodies).

In the AuNPsCM group, the intima was severely reduced in thickness, mainly due to the presence of a very thin, uniform subendothelial connective layer (Figure 11A,B). The endothelial cells had a normal ultrastructure (with many vesicles of transcytosis and Weibel–Palade bodies), and sometimes were prominent into the lumen (Figure 11A,B). All of these features suggested a previous denudation of the inner lamina. The media showed smooth muscle cells with normal aspect, connected by a homogeneous extracellular matrix (Figure 11C,D). All elastic laminae were homogeneous (Figure 11A–D).

**Figure 12:**
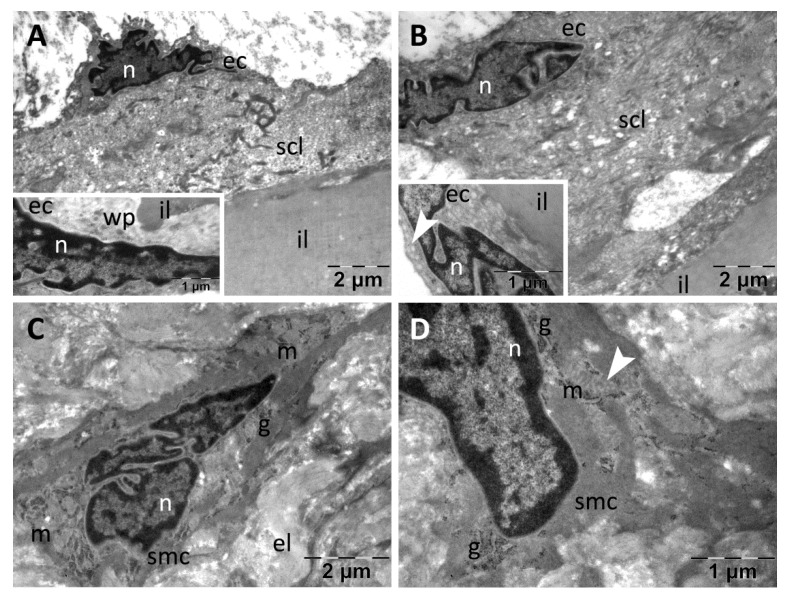
TEM investigation of the descending aorta in rats with HFD for 9 months; the last month with experimental-induced diabetes mellitus and oral administration of *Cornus mas* L. extract showed almost normal ultrastructure in both intima that contained a heterogeneous subendothelial connective layer (**A**,**B**), and media (**C**,**D**) (arrowhead, vesicles of endocytosis in muscle cells and of transcytosis in endothelial cells; ec, endothelial cell; el, elastic lamina; il, inner elastic lamina; g, glycogen; m, mitochondria; n, nucleus; scl, subendothelial connective layer; wp, Weibel–Palade bodies).

In the aortas from the CM group, the intima was well represented: the endothelial cells had normal aspect and ultrastructure while the subendothelial connective layer was thick but heterogeneous, showing rarefied regions and regions consisting of compact packed, dense fibers (Figure 12A,B). The media contained smooth muscle cells with characteristic aspect, and the extracellular matrix was homogeneous (Figure 12C,D). All elastic laminae were homogeneous (Figure 12A–D).

## 4. Discussion

The present study investigated the modifications in the aorta wall structure and functionality in rats with 9 months of a high-fat diet, the last month with experimental-induced diabetes mellitus and treatment.

To evaluate the effects of gold nanoparticles functionalized with *Cornus mas* L. extract, insulin and pioglitazone were used as positive controls and CMC as a negative control. The effect of *Cornus mas* L. simple extract administration on this pathological condition (diabetes mellitus) was also studied.

The imaging examination of the descending aorta in a length of 1 cm, right after the aortic arch, showed significant modifications among the groups.

Insulin administration produces vasodilation [19] through its direct action on the endothelial cells that release nitric oxide [20]. Insulin also has anti-atherosclerotic effects [21] that are blocked in DM by the release of the protein kinase C isoforms, β and δ, in several tissues but also in the aorta and heart [22]. In the present study, similar to these previous researches, significant aorta vasodilation with significant increases in aorta volume was observed in diabetic rats that received insulin as a treatment, but with non-significant decreases in blood flow velocity. The TEM investigation identified ultrastructural modifications in the intima (thin endothelium with vacuolized endothelial cells, few of these cells were removed and replaced by small clots; heterogenous subendothelial connective layer with macrophages and vacuoles) and in media (several smooth muscle cells with vacuoles, probably the enlarged mitochondria). Compared to the aorta in rats with DM and prolonged HFD treated with CMC that presented the modifications specific for this pathological condition in the intima (endothelial cells with vacuoles, only a few partially detached; heterogenous subendothelial connective layer with macrophages) and in media (smooth muscle cells with endocytic vesicles and large vacuoles), the aorta of the rats treated with insulin presented much altered ultrastructure. Our findings are concordant with the data presented by Kaur et al. in their review that identified hyperglycemia as a trigger for endothelial dysfunction, platelet activation and adhesion to the wounded vessel wall area [23]. The variable thickness of the aorta subendothelial connective layer, found in our rats with insulin treatment, may explain the aorta stiffness presented by Dec-Gilowska et al. in their study of patients with diabetes mellitus type 2; stiffness that was much higher in patients that received insulin [24]. The accumulation of macrophages in the intima of the aorta found in our TEM investigation may be, as Quinn showed in her study, the consequence of hyperglycemia that stimulates the low density lipoprotein (LDL) glycation, process that makes them miss the connection with LDL receptors, leading to the cholesteryl esters synthesis, a mechanism that attracts the macrophages to uptake these lipid molecules and to develop the foam cells [2]. The presence of foam cells in the aorta wall of our rats could be also the result of the prolonged HFD that transformed the smooth muscle cells of the aorta into macrophage-like cells for lipids storage, as Gui et al. presented in their review [25]. The well-developed mitochondria observed in the smooth muscle cells might be the effect of insulin administration, a result that is in concordance with those mentioned by Karwi et al. in their study performed on isolated mouse hearts, using insulin to analyze glucose oxidation [26].

Pioglitazone administration had similar effects to the insulin treatment. The ultrasound examination of the rats’ aorta treated with this antidiabetic medication that proved to also have antioxidant and anti-inflammatory effects [11,27], had the most significant vasodilation with the lowest significant blood flow velocity, compared to the rats in the negative control group. These modifications were confirmed with the MRI investigation that recorded the highest descending aorta volume (Figure 7). These findings were concordant with the study performed on persons with impaired glucose regulation by Yu et al., which showed the increase in nitric oxide levels after the administration of pioglitazone at a dose of 15 mg/day for 12 weeks [28]. The vasodilation may be also explained by the opening effect of pioglitazone on the smooth muscle K_V_ (voltage-dependent K^+^) and/or KIR (inward rectifier K^+^) channels that was reported by Nomura et al. in their study on the isolated rat aorta [29]. The TEM investigation on the aorta wall of the Pioglitazone group showed similar effects as were recorded in the Insulin group, no clots inside the endothelial layer but Weibel–Palade bodies inside the endothelial cells, the sign of DM modifications of these cells toward a secretory phenotype that leads to atheroma development, as Toma et al. mentioned in their review [30].

Gold nanoparticles functionalized with *Cornus mas L.* extract (AuNPsCM) altered the aorta wall in a specific manner: it stimulated the endothelial layer to produce Weibel–Palade bodies and thinned the subendothelial connective layer. The presence of Weibel–Palade bodies in high amounts in the endothelial cells was correlated with the initial phase of DM, before the onset of the vascular alterations [31]. The fact that the endothelial layer was not affected by the AuNPsCM treatment might be explained by the small dimensions of these delivery systems (19 nm) that permitted their passage toward the subendothelial layer. The denudation of the inner lamina and the subendothelial connective layer alteration that were observed in the aorta of the diabetic rats with HFD treated with AuNPsCM were also noticed in our preliminary study, performed on rats with only HFD [16], suggesting that these nanoparticles were involved in the ultrastructural modifications of these areas. Compared to the CMC (negative control) group, the ultrasound examination showed a significant increase in the descending aorta diameter, a significant decrease in blood flow velocity, and the MRI investigation presented a significant increase in this aorta segment volume, results that correlate with TEM findings: the thinning of the aorta wall might be the cause of the diameter increase.

The administration of *Cornus mas* L. extract as a simple solution improved the aorta wall ultrastructural aspect in rats with DM and prolonged HFD, compared to the negative and positive control groups, while the imaging investigations showed similar results to the negative control. The beneficial effects of natural extract might be explained by its chemical compounds that we identified through HPLC determination: flavonoids (naringenin, kaempferol, rutoside and their derivatives) that have been previously presented in the literature as part of this natural compound [32,33,34]; phenolic acids (caffeic and chlorogenic acids) mentioned by Bayram and Ozturkcan [35]; but also chrysin, hyperoside and luteolin. Several studies presented caffeic acid as an antioxidant [36], anti-inflammatory [37] and even a smooth muscle relaxing compound, as Siva et al. showed in their study performed in organ bath using the rat thoracic aorta [38]. The other phenolic acid identified in *Cornus mas* L. extract, chlorogenic acid, was found in our previous studies as an efficient antioxidant and anti-inflammatory element, in a dose-dependent manner [39,40], Wu et al. described this natural compound as anti-atherosclerotic [41], Hada et al. as a potent inhibitor of aorta senescence in their study performed on mice with saline or angiotensin II administration [42], and many other studies indicated the beneficial effects of this acid. Among the *Cornus mas* flavonoids identified in our study, naringenin was presented by Fallahi et al. in their experiment performed on diabetic rats as a factor that might improve the endothelial function of the aorta [43], kaempferol was described by Xiao et al. in their study realized in apolipoprotein E-deficient mice as an inhibitor of the aorta oxidative stress and atherosclerotic lesion [44] while Ren et al. in their review, exposed this flavonoid properties in DM alleviation [45]. In their study performed on mice with HFD and streptozotocin-induced DM, Lee et al. found that rutin (rutoside) could improve the activity of the β-cell function [46]. Chrysin, the flavonoid found in our HPLC determination, has anti-atherosclerotic effects through lipid peroxidation inhibition, as Farkhondeh et al. presented in their review [47]; produces vasodilation, an effect related by Tew at al. in their experiment performed on rat aorta ring [48], and like luteolin (another identified flavonoid in *Cornus mas* L. extract), may restore the vascular response [49]. Luteolin has protective effects on β-cells, providing protection against the noxious mechanisms that occur in DM and may improve endothelial dysfunction, as Queiroz et al. revealed in their experiment using the administration of this natural compound in rats (10 mg/kg/day for 2 months) [50]. Luteolin decreases the oxidative stress protecting the aorta function, as Qian et al. showed in their study on aorta rings of male Sprague Dawley rats [51] and may have antioxidant, anti-inflammatory and antidiabetic effects [52]. In our experiment, we also identified hyperoside as a *Cornus mas* constituent, a chemical that improves endothelial dysfunction and protects the myocardial cells in diabetes mellitus, properties that were presented by Xu et al. in their review [53]. All of these beneficial effects of the elements identified in *Cornus mas* L. extract may explain our result: the preservation of the aorta wall function and structure, in experimental-induced DM in rats with chronic HFD.

The present study focused on the modifications that might occur in the wall of the descending aorta in rats with prolonged HFD and experimental-induced DM when insulin, pioglitazone or *Cornus mas* L. extract in two forms (simple or nanoparticulate solutions) were administered as a daily treatment for one month. The results showed the beneficial effects of *Cornus mas* L. simple solution on the intima of the diabetic aorta wall with a normal aspect of endothelial cells, and the injury produced by the AuNPsCM treatment on the subendothelial connective layer.

The aim of our larger project was to evaluate the effects of gold nanoparticles suspended in citrate buffer (AuNPs) (Sigma Aldrich, Germany) or functionalized with *Cornus mas* L. extract (AuNPsCM) on the aorta wall in healthy rats [54], rats with prolonged HFD [16] and also rats with chronic HFD and experimental-induced DM ([11] and the current study).

All of the results of the present study are concordant with our previous experiments and may be used to develop an adjuvant for DM treatment, based on a simple solution of *Cornus mas* L. extract.

## 5. Conclusions

Gold nanoparticles functionalized with the bioactive compounds from *Cornus mas* L. extract produced aorta wall alterations with effects on blood flow. *Cornus mas* L. extract administered as a simple solution improved the aorta wall ultrastructure and preserved its function.

## Figures and Tables

**Table 1:** LC/MS identification and quantification of polyphenols from the tested *Cornus mas* L. extract.

Identified Compounds	Reference		Sample		
	Retention Time (min)	Main MS Transition	Retention Time (min)	Main MS Transition	Content (mg/mL)
Caffeic acid	13.5	179.0 > 135.0	13.7	179.0 > 135.0	0.167 ± 0.0287
Chlorogenic acid	11.9	353.0 > 191.0	12.4	353.0 > 191.0	0.019 ± 0.0053
Chrysin	29.7	253.0 > 143.0	30.0	253.0 > 143.0	0.011 ± 0.0012
Hyperoside	20.3	463.1 > 300.0	20.1	463.1 > 300.0	0.010 ± 0.0005
Kaempferol	27.9	285.0 > 187.0	28.6	285.0 > 187.0	0.004 ± 0.0005
Luteolin	26.8	287.0 > 153.0	26.6	287.0 > 153.0	0.006 ± 0.0008
Naringenin	26.2	271.0 > 119.0	27.5	271.0 > 119.0	0.011 ± 0.0012
Rutoside	20.2	609.0 > 300.0	20.0	609.0 > 300.0	0.021 ± 0.0033

Flavonoids and phenolic acids were found to be the main classes of the compounds identified in *Cornus mas* L. extract.

## Data Availability

Data is contained within the article.
